# Combined Vision and Hearing Difficulties Results in Higher Levels of Depression and Chronic Anxiety: Data From a Large Sample of Spanish Adults

**DOI:** 10.3389/fpsyg.2020.627980

**Published:** 2021-01-18

**Authors:** Shahina Pardhan, Lee Smith, Rupert Bourne, Adrian Davis, Nicolas Leveziel, Louis Jacob, Ai Koyanagi, Guillermo F. López-Sánchez

**Affiliations:** ^1^Faculty of Health, Education, Medicine and Social Care, School of Medicine, Vision and Eye Research Institute, Anglia Ruskin University, Cambridge, United Kingdom; ^2^Cambridge Centre for Sport and Exercise Sciences, Anglia Ruskin University, Cambridge, United Kingdom; ^3^ENT and Audiology, Imperial College London, London, United Kingdom; ^4^Research and Development Unit, Parc Sanitari Sant Joan de Déu, CIBERSAM, Barcelona, Spain; ^5^Faculty of Medicine, University of Versailles Saint-Quentin-en-Yvelines, Montigny-le-Bretonneux, France; ^6^ICREA, Barcelona, Spain

**Keywords:** vision impairment, hearing impairment, depression, anxiety, sensory impairment

## Abstract

**Objective:**

Individually, vision and hearing impairments have been linked to higher levels of anxiety and depression. We investigated the effect of dual sensory impairment (difficulty seeing and hearing) in a large representative sample of Spanish adults.

**Methods:**

Data from a total of 23,089 adults (age range: 15–103 years, 45.9% men) from the Spanish National Health Survey 2017 were analyzed. Self-reported difficulty of seeing and hearing (exposures), and depression and chronic anxiety (outcomes) were analyzed. Multivariable logistic regression was assessed for difficulty with vision alone, hearing alone and with difficulty with both, adjusting for gender, age, marital status, living as a couple, education, smoking, alcohol consumption, BMI, physical activity, use of glasses/contact lenses, and hearing aid.

**Results:**

Visual difficulty, hearing difficulty, and dual difficulties were all associated with significantly higher odds for depression (ORs 2.367, 2.098, and 3.852, respectively) and for chronic anxiety (ORs 1.983, 1.942, and 3.385, respectively). Dual sensory difficulty was associated with higher odds ratios for depression and anxiety when compared to either impairment alone.

**Conclusion:**

Dual sensory difficulty is associated with significantly higher odds of anxiety and depression when compared to either vision or hearing difficulty alone. Appropriate interventions are needed to address any reversible causes of vision and hearing as well as anxiety and depression in people in these specific groups.

## Introduction

Depression and anxiety are among the most prevalent mental health disorders globally. Depression affects around 264 million people globally, and is characterized by persistent sadness, lack of interest or pleasure in previously rewarding or enjoyable activities, problems with sleep and appetite, tiredness and poor concentration (American Psychiatric Association, 2020; Centers for Disease Control and Prevention, 2020; World Health Organization, 2020a,b). Anxiety affects around 284 million people worldwide and is typically characterized by apprehension (worries about future misfortunes, feeling “on edge,” difficulty in concentrating, etc.), motor tension (restless fidgeting, tension headaches, trembling, and inability to relax) and autonomic overactivity (light-headedness, sweating, tachycardia or tachypnea, epigastric discomfort, dizziness, dry mouth, etc.) (American Psychiatric Association, 2017; Ritchie and Roser, 2018). Also, depression and anxiety have been associated with higher incidence of suicide attempts (Casey et al., 2006; Nepon et al., 2010), reduced sleep (Alvaro et al., 2013), cardiovascular disease (Van der Kooy et al., 2007; Batelaan et al., 2016), cancer risk (Oerlemans et al., 2007; Shen et al., 2013), and mortality (Schulz et al., 2000; Mykletun et al., 2009).

Furthermore, depression has also been linked to sensory losses such as vision and hearing. For example, in one large representative sample of Korean adults, it was found that the risk of depression increased significantly in people who had visual impairment (adjusted HR = 1.15, *P* = 0.036) and also in those who were blind (adjusted HR = 1.31, *P* = 0.016) (Choi et al., 2018). Other studies (Zhang et al., 2013; Ribeiro et al., 2015; van der Aa Hilde et al., 2015; Nollett et al., 2019) have found similar findings, obtaining also significant associations between visual impairment and depression. Links between visual impairment and anxiety shows mixed evidence with some studies suggesting little to no association (Evans et al., 2007; Yochim et al., 2012), whilst other studies show some association (Kempen et al., 2012). Hearing impairment has also been found to be associated with anxiety and depression (Bernabei et al., 2011; Li et al., 2014; Contrera et al., 2017). Systematic reviews and meta-analysis demonstrate significantly higher odds of depression in older adults with hearing losses (Adigun, 2017; Lawrence et al., 2020).

It is likely that the association between sensory impairment and mental health is bidirectional, and this could be explained by several factors associated to both sensory impairment and mental health, including difficulties with socializing (Papadopoulos et al., 2011; Ritchie and Roser, 2018; World Health Organization, 2020a,b), activities of daily living (Pérès et al., 2017; Ritchie and Roser, 2018; World Health Organization, 2020a,b), inadequate nutrition (Jones and Bartlett, 2018; Ritchie and Roser, 2018; World Health Organization, 2020a,b), and reduced levels of physical activity (Ritchie and Roser, 2018; Smith et al., 2019; López-Sánchez et al., 2020b; World Health Organization, 2020a,b). In this study we treated difficulty seeing and hearing as the exposures and depression and anxiety as the outcomes.

Previous studies have analyzed the association between visual impairment alone and hearing impairment alone in depression or anxiety. However, importantly, no study to date has compared the risk of dual vision and hearing visual difficulties (the two sensory impairments at the same time in the same person) with both depression and anxiety, although several systematic reviews provide risk ratios for vision and hearing losses individually. Moreover, as studies cited in the literature are from different countries, direct comparison of vision or hearing losses becomes difficult as environmental and healthcare availability may vary in different countries and this may impact on prevalence rates of depression and anxiety. It is therefore important to carry out studies in different countries. There may be local environmental and healthcare differences in various regions of Spain but there are many country-wide characteristics in Spain that makes important to analyze the Spanish population as a whole in order to allow comparisons with other countries. In addition, it is likely that a stronger association will exist with people with dual difficulty since these individuals will struggle more with relation to day-to-day activities. We aim to investigate, using cross-sectional associations, the risk of anxiety and depression in people with seeing difficulty only, hearing difficulty only and difficulties with both seeing and hearing in a large representative sample of Spanish adults. This is the first study about this topic in Spain and is particularly important as previous data suggests a high prevalence of difficulty seeing (12.1%) and difficulty hearing (12.7%) among Spanish adults (Latorre-Arteaga et al., 2017), which are higher than those found in other countries. For example, the prevalence of visual impairment among 14,687 adults in Germany was 0.37% and the estimated prevalence of people with disabling hearing loss in the world is 6.1% (Wolfram et al., 2019; World Health Organization, 2020c). Furthermore, the prevalence of depression and anxiety in Spain is also high in comparison with other countries. For example, Ramón-Arbués et al., found a prevalence of depression of 18.4% and a prevalence of anxiety of 23.6% in Spanish adults, percentages that are higher than those found in adults in India (depression 12.1% and anxiety 19.0%) or in Brazilian adults (depression 14%) (Sahoo and Khess, 2010; Silva et al., 2014; Ramón-Arbués et al., 2020).

## Methods

### The Survey

Spanish National Health Survey (year 2017) was conducted between October 2016 and October 2017. A detailed description of the Spanish National Health survey is provided in previous literature (Ministerio de Sanidad, Consumo y Bienestar Social & Instituto Nacional de Estadística, 2017a, b) and we provide only a brief overview here. A three stage stratified sampling method was employed for data collection. In the first stage, census sections were taken into consideration, in the second stage the family dwellings, and in the third stage an adult (older than 15 years) was chosen within each dwelling. The probability of selecting a section in each stratum was dependent on its size. A dwelling was chosen with equal probability in each section using systematic sampling. Samples were therefore self-weighted in each stratum. The random Kish method was applied to select the person who participated in the Adult Questionnaire, thereby assigning the same probability to all adults older than 15 years living in the house. The inclusion criteria were residents of Spain, being older than 15 years and not have mental incapacity. The exclusion criteria were not residing in Spain, not being older than 15 years or having mental incapacity. A total of 23,089 adults (age range: 15–103 years) participated in this survey, allowing for a representative sample of the adult Spanish population. CAPI (computer-assisted personal interviewing) method was used for the data collection, and data were collected in the household of the participants. Trained interviewers completed the questionnaires with the responses of the participants. The questionnaire was conducted in Spanish. Informed consent form was signed by all participants before responding to the questionnaire. A total of 23089 adults (94.80%) completed the questionnaire, 317 adults did not want to participate (1.30%), 937 adults were not at home (3.85%) and 13 adults (0.05%) had mental incapacity and could not complete the questionnaire. This research was conducted in accordance with the Declaration of Helsinki of the World Medical Association. In accordance with the regulation of the European Union, the file data for public use does not require the approval of an accredited ethics committee for statistical or research purposes.

### Difficulty Seeing and Hearing (Exposure)

An affirmative answer to the question “Do you have difficulty seeing?” were noted. This group comprised participants who reported difficulty seeing but did not habitually use any visual correction (spectacles/contact lenses) or had difficulty seeing even with their habitual glasses/contact lenses. Self-reported vision impairment is a method widely used and accepted in epidemiological papers to measure vision impairment (Steinman and Allen, 2012; Liljas et al., 2017; Frank et al., 2019; Xiang et al., 2020).

People who answered affirmatively to the question “Do you have difficulty hearing what is being said in a conversation with another person in a quiet place?” were considered to have difficulty hearing. This group was composed of people who had difficulty hearing with or without the using a hearing aid. Self-reported hearing loss is a valid method to measure hearing loss (Sindhusake et al., 2001).

Those who answered affirmatively to both hearing and vision difficulties were categorized into dual difficulty group.

### Depression and Chronic Anxiety (Outcome)

Participants who responded affirmatively to “Have you ever been diagnosed of depression by a physician?”; “Have you ever been diagnosed of chronic anxiety by a physician?” were considered to have depression and chronic anxiety, respectively. The question for depression has been previously validated (Sanchez-Villegas et al., 2008). The question for anxiety is not validated but it has been used in previous scientific literature (Jacob et al., 2020a,b, c; López-Bueno et al., 2020; López-Sánchez et al., 2020a).

### Covariates

The selection of the other control variables was based on bivariate analyses and on past literature (Bonnet et al., 2005; Zoeller, 2007; Acar et al., 2011; Mendieta Mayorga, 2012; Fazzi et al., 2015; McCusker and Koola, 2015; López-Sánchez et al., 2019). Sociodemographic variables included gender, age, marital status, living as a couple and education. Age was categorized according to the accepted international definition of older adults as: (1) <65 years and (2) ≥65 years (Orimo et al., 2006). Marital status was categorized as: (1) Married, (2) Single, and (3) Widowed/divorced/separated. Living as a couple was categorized by yes or no. Education was based on the highest educational level achieved and was categorized as: (1) No formal education, (2) Primary, (3) Secondary, and (4) Tertiary. Smoking status was self-reported and categorized as never, current smoker, and past smoker. Alcohol consumption in the last 12 months was self-reported and categorized as: (1) Daily or almost daily, (2) 5–6 days per week, (3) 3–4 days per week, (4) 1–2 days per week, (5) 2–3 days in a month, (6) Once a month, (7) Less than once a month, (8) Not in the last 12 months, I have stopped drinking alcohol, and (9) Never or just a few sips to try it throughout life. Height and weight were self-reported. Body mass index (BMI) was calculated as weight in kilograms divided by height in meters squared, and it was categorized according to World Health Organization^1^ as: (1) Underweight, (2) Normal, (3) Overweight, and (4) Obesity. The International Physical Activity Questionnaire (IPAQ) Short Form was used to measure physical activity. The unit of physical activity used was MET-minutes/week, where MET is the Metabolic Equivalent of Task. Total physical activity MET-minutes/week were calculated through the following formula: sum of Walking + Moderate + Vigorous MET-minutes/week scores (IPAQ group, 2005). Participants were divided in two categories according to the guidelines for data processing and analysis of the IPAQ (IPAQ group, 2005): (1) fewer than 600 MET-minutes/week and (2) at least 600 MET-minutes/week, equivalent to meeting current physical activity recommendations. The age group of adults ≥70 years did not complete the IPAQ Short Form, as this questionnaire was developed for population surveillance of physical activity among adults aged 15-69 years, and its use with older and younger age groups is not recommended (IPAQ group, 2005). IPAQ has been validated in adult populations from different countries showing acceptable validity (ρ = 0.30, 95% CI: 0.23–0.36) and reliability (Spearman’s ρ = 0.81, 95% CI: 0.79–0.82) (Craig et al., 2003; Rodriguez-Munoz et al., 2017). Participants who responded affirmatively to “Have you ever been diagnosed with cataracts?” were considered to have cataracts. Participants who responded affirmatively to the questions “Do you use glasses or contact lenses” and “Do you use hearing aid?” were considered to use glasses or contact lenses and hearing aid, respectively.

### Statistical Analysis

The statistical analysis was performed with SPSS 23.0 (IBM, Armonk, NY, United States). The outcomes: prevalence (frequencies and percentages) of depression and chronic anxiety in Spanish adults by exposure: difficulties seeing and hearing and all other covariates is shown in Table 1. Those covariates that were significant with chi-square tests were included in the regression models (Table 2). All covariates were significant and, therefore, included in the two models (depression and anxiety), except hearing aid, which was included in the model to predict depression but not in the model to predict chronic anxiety because it was not significant in predicting chronic anxiety. Multivariable logistic regression analysis assessed the association between difficulties seeing and hearing and other covariates with depression or anxiety (mental health outcomes). Goodness-of-fit and diagnostic tests were conducted in order to check the suitability of the regression model. The models were adjusted for gender, age, marital status, living as a couple, education, smoking, alcohol, BMI, physical activity, glasses/contact lenses, and hearing aid (this last one only in the model to predict chronic anxiety). All variables were included in the models as categorical variables. Results from the logistic regression analyses are presented as odds ratios (ORs) with 95% confidence intervals (CIs). The missing data were the following: marital status (*n* = 39; 0.17%), living as a couple (*n* = 139; 0.60%), smoking (*n* = 22; 0.10%), alcohol consumption (*n* = 26; 0.11%), BMI (*n* = 1070; 4.63%), physical activity (*n* = 5,312; 23.01%), hearing aid (*n* = 1; 0.004). Complete-case analysis was carried out. The level of statistical significance was set at *p* < 0.05.

**TABLE 1:** Prevalence of chronic anxiety and depression in Spanish adults, by difficulties seeing and hearing and by covariates.

**Variables**	**Categories**	**Depression**	**Chronic anxiety**
Overall (*n* = 23089)	–	2464 (10.7)	2078 (9.0)
Only difficulty seeing*^†^	Yes (*n* = 2550)	520 (20.4)	397 (15.6)
	No (*n* = 20539)	1944 (9.5)	1681 (8.2)
Only difficulty hearing*^†^	Yes (*n* = 1607)	280 (17.4)	192 (11.9)
	No (*n* = 21482)	2184 (10.2)	1886 (8.8)
Difficulty seeing and hearing*^†^	Yes (*n* = 908)	263 (29.0)	179 (19.7)
	No (*n* = 22181)	2201 (9.9)	1899 (8.6)
Difficulty seeing or hearing*^†^	Yes (*n* = 5065)	1063 (21.0)	768 (15.2)
	No (*n* = 18024)	1401 (7.8)	1310 (7.3)
Gender*^†^	Females (*n* = 12494)	1816 (14.5)	1510 (12.1)
	Males (*n* = 10595)	648 (6.1)	568 (5.4)
Age*^†^	≥65 years (*n* = 7023)	1163 (16.6)	724 (10.3)
	<65 years (*n* = 16066)	1301 (8.1)	1354 (8.4)
Marital status*^†^	Single (*n* = 5888)	403 (6.8)	409 (6.9)
	Married (*n* = 12465)	1377 (11.0)	1193 (9.6)
	Widowed/divorced/separated (*n* = 4697)	681 (14.5)	473 (10.1)
Living as a couple*^†^	No (*n* = 10475)	1385 (13.2)	1087 (10.4)
	Yes (*n* = 12475)	1071 (8.6)	982 (7.9)
Education*^†^	No formal education (*n* = 2742)	545 (19.9)	356 (13.0)
	Primary (*n* = 4464)	706 (15.8)	506 (11.3)
	Secondary (*n* = 9936)	908 (9.1)	868 (8.7)
	Tertiary (*n* = 5947)	305 (5.1)	348 (5.9)
Smoking*^†^	Current smoker (*n* = 5398)	565 (10.5)	565 (10.5)
	Past smoker (*n* = 5962)	573 (9.6)	508 (8.5)
	Never (*n* = 11707)	1325 (11.3)	1005 (8.6)
Alcohol*^†^	Daily or almost daily (*n* = 3771)	330 (8.8)	267 (7.1)
	5–6 days per week (*n* = 355)	19 (5.4)	13 (3.7)
	3–4 days per week (*n* = 801)	64 (8.0)	48 (6.0)
	1–2 days per week (*n* = 3424)	196 (5.7)	215 (6.3)
	2–3 days in a month (*n* = 2441)	140 (5.7)	149 (6.1)
	Once a month (*n* = 1484)	120 (8.1)	120 (8.1)
	Less than once a month (*n* = 2527)	260 (10.3)	258 (10.2)
	Not in the last 12 months, I have stopped drinking alcohol (*n* = 3545)	650 (18.3)	502 (14.2)
	Never or just a few sips to try it throughout life (*n* = 4715)	683 (14.5)	504 (10.7)
BMI*^†^	Underweight (*n* = 475)	48 (10.1)	48 (10.1)
	Normal (*n* = 9301)	748 (8.0)	671 (7.2)
	Overweight (*n* = 8333)	917 (11.0)	772 (9.3)
	Obesity (*n* = 3910)	597 (15.3)	468 (12.0)
Physical activity*^†^	<600 MET-minutes/week (*n* = 5366)	680 (12.7)	657 (12.2)
	≥600 MET-minutes/week (*n* = 12411)	897 (7.2)	886 (7.1)
Cataracts*^†^	Yes (*n* = 2878)	681 (23.7)	418 (14.5)
	No (*n* = 20211)	1783 (8.8)	1660 (8.2)
Glasses/contact lenses*^†^	Yes (*n* = 15629)	1982 (12.7)	1627 (10.4)
	No (*n* = 7443)	476 (6.4)	448 (6.0)
Hearing aid*	Yes (*n* = 957)	145 (15.2)	90 (9.4)
	No (*n* = 22108)	2311 (10.5)	1982 (9.0)

Values expressed in Frequencies (Valid %). Significant differences between groups were calculated with chi-square tests. *Significant differences in the prevalence of depression. ^†^ Significant differences in the prevalence of chronic anxiety.

*Those covariates that were significant were included in the regression models (Table 2). All covariates were included in the two models (depression and anxiety), except hearing aid, which was included in the model to predict depression but not in the model to predict chronic anxiety.*

**TABLE 2:** Associations between difficulties hearing and seeing (exposures) and other covariates with chronic anxiety and depression (outcomes) in Spanish adults, estimated by multivariable logistic regression.

**Variables**	**Categories**	**Depression^a^**	**Chronic anxiety^b^**
Only difficulty seeing	Yes	2.367 (2.035–2.754)***	1.983 (1.698–2.315)***
	REF: No	1.0	1.0
Only difficulty hearing	Yes	2.098 (1.667–2.640)***	1.942 (1.537–2.455)***
	REF: No	1.0	1.0
Difficulty seeing and hearing	Yes	3.852 (2.725–5.444)***	3.385 (2.377–4.821)***
	REF: No	1.0	1.0
Gender	Females	2.357 (2.069–2.684)***	2.250 (1.981–2.556)***
	REF: Males	1.0	1.0
Age	≥65 years	1.113 (0.936–1.323)	1.319 (1.089–1.597)**
	REF: <65 years	1.0	1.0
Marital status	Single	0.688 (0.569–0.831)***	0.771 (0.639–0.930)**
	Married	0.961 (0.820–1.126)	0.995 (0.847–1.169)
	Widowed/divorced/separated	1.0	1.0
Living as a couple	No	1.672 (1.491–1.874)***	1.430 (1.278–1.601)***
	REF: Yes	1.0	1.0
Education	No formal education	2.166 (1.693–2.772)***	1.887 (1.474–2.417)***
	Primary	2.310 (1.917–2.782)***	1.781 (1.483–2.139)***
	Secondary	1.676 (1.437–1.955)***	1.441 (1.248–1.664)***
	REF: Tertiary	1.0	1.0
Smoking	Current smoker	0.606 (0.527–0.697)***	0.566 (0.493–0.648)***
	Past smoker	0.733 (0.633–0.850)***	0.675 (0.583–0.781)***
	REF: Never	1.0	1.0
Alcohol	Daily or almost daily	0.811 (0.659–0.999)*	0.829 (0.672–1.022)
	5–6 days per week	0.550 (0.306–0.991)*	0.483 (0.258–0.906)*
	3–4 days per week	0.878 (0.631–1.221)	0.699 (0.491–0.994)*
	1–2 days per week	0.614 (0.496–0.759)***	0.748 (0.611–0.915)**
	2–3 days in a month	0.553 (0.435–0.703)***	0.639 (0.507–0.805)***
	Once a month	0.779 (0.602–1.009)	0.914 (0.714–1.169)
	Less than once a month	0.845 (0.689–1.037)	1.010 (0.829–1.231)
	Not in the last 12 months, I have stopped drinking alcohol	1.613 (1.355–1.920)***	1.404 (1.173–1.680)***
	REF: Never or just a few sips to try it throughout life	1.0	1.0
BMI	Underweight	0.647 (0.440–0.951)*	0.807 (0.563–1.157)
	Normal	0.579 (0.496–0.676)***	0.649 (0.556–0.758)***
	Overweight	0.845 (0.730–0.980)*	0.892 (0.768–1.036)
	REF: Obesity	1.0	1.0
Physical activity	<600 MET-minutes/week	1.497 (1.334–1.681)***	1.486 (1.326–1.667)***
	≥600 MET-minutes/week	1.0	1.0
Cataracts	Yes	1.760 (1.418–2.185)***	1.453 (1.151–1.834)**
	REF: No	1.0	1.0
Glasses/contact lenses	Yes	1.663 (1.456–1.900)***	1.686 (1.479–1.920)***
	REF: No	1.0	1.0
Hearing aid	Yes	1.017 (0.724–1.429)	–
	REF: No	1.0	–

Values expressed in Odds Ratio (95% Confidence Interval). *P < 0.05, **P < 0.01, ***P < 0.001. REF, reference category.

*^*a*^Model adjusted for gender, age, marital status, living as a couple, education, smoking, alcohol, BMI, physical activity, cataracts, glasses/contact lenses, and hearing aid. ^*b*^Model adjusted for gender, age, marital status, living as a couple, education, smoking, alcohol, BMI, physical activity, cataracts, and glasses/contact lenses.*

## Results

**FIGURE 1:**
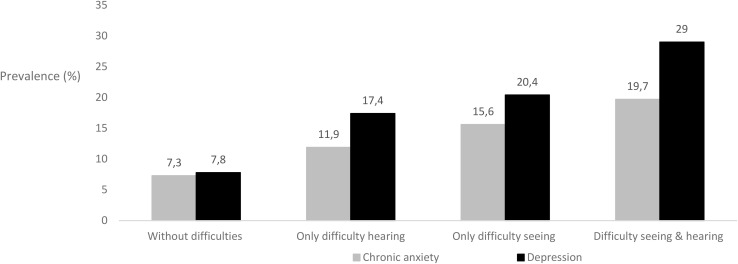
Prevalence of depression and chronic anxiety in Spanish adults with seeing and hearing difficulties.

The data comprised 23,089 Spanish participants aged 15–103 years (mean age 53.4 ± 18.9 years) with roughly equal gender representation (45.9% men). The prevalence of depression in the total sample was 10.7%. Analyzing separate groups, the prevalence of depression was 7.8% in the sample without any difficulties, 20.4% in those with seeing difficulty, 17.4% in those with hearing difficulty and 29% in those with both seeing and hearing difficulties. The overall prevalence of chronic anxiety in the whole sample was 9%, with 7.3% in the those without either seeing/hearing difficulties, 15.6% in those with seeing difficulty, 11.9% in the population with hearing difficulty and 19.7% in those with both seeing and hearing difficulties (Table 1 and Figure 1).

In analyzing the prevalence of depression by covariates, it was observed that the following groups had the highest prevalence of depression in each variable: females (14.5%), ≥65 years (16.6%), widowed/divorced/separated (14.5%), not living as a couple (13.2%), no formal education (19.9%), those who never smoked (11.3%), those who did not drink alcohol in the last 12 months (18.3%), obesity (15.3%), <600 MET-minutes/week of physical activity (12.7%), cataracts (23.7%), glasses/contact lenses (12.7%) and hearing aid (15.2%).

When analyzing the prevalence of chronic anxiety by covariates, the following groups had the highest prevalence of chronic anxiety in each variable: females (12.1%), ≥65 years (10.3%), widowed/divorced/separated (10.1%), not living as a couple (10.4%), no formal education (13.0%), current smokers (10.5%), those who did not drink alcohol in the last 12 months (14.2%), obesity (12.0%), <600 MET-minutes/week of physical activity (12.2%), cataracts (14.5%), glasses/contact lenses (10.4%) and hearing aid (9.4%) (Table 1).

Multivariable logistic regression showed that seeing difficulty, hearing difficulty and the combination of both were associated with significantly higher odds for depression (ORs 2.367, 2.098, and 3.852, respectively) and also for chronic anxiety (ORs 1.983, 1.942, and 3.385, respectively) (Table 2). With regards to the associations between covariates and depression or chronic anxiety, the population groups with a significantly higher risk of depression and chronic anxiety were females, widowed/divorced/separated, those not living as a couple, those with no tertiary education, those who never smoked, those who did not drink alcohol in the last 12 months, those with obesity, people doing less physical activity, those with cataracts and those with glasses or contact lenses (Table 2).

The suitability of the regression models was assessed. The final model to predict depression was a significant improvement in fit over the null model [χ^2^(29) = 1,311.771, *p* < 0.001] and the deviance chi-square indicated good fit [χ^2^(8557) = 6,139.018, *p* = 1.000]. For the model to predict chronic anxiety, the final model was a significant improvement in fit over the null model [χ^2^(28) = 907,403, *p* < 0.001] and the deviance chi-square indicated good fit [χ^2^(8352) = 6,050.613, *p* = 1.000].

## Discussion

In this large representative sample of Spanish adults, seeing difficulty and hearing difficulty were both associated with significantly higher odds for depression and anxiety, with seeing difficulty showing a slightly higher risk for both mental health indices. However, in people with dual sensory difficulties (both vision and hearing difficulties together) the odds were significantly higher. This is the first study to show that people with dual vision and hearing visual difficulties (the two sensory impairments at the same time in the same person) have a higher risk of both depression and anxiety. It is also the first study showing this effect in a Spanish population.

Our data compare well with previous studies that have analyzed the association of visual impairment alone and hearing impairment alone with depression and anxiety. A systematic review, based on 35 papers and 147,148 people reported similar odds ratios (1.54, 95% CI = 1.31-1.80) for people with hearing difficulties (Lawrence et al., 2020). In people with eye diseases, Zheng et al. review (Zheng et al., 2017), based on 28 papers, reported similar odds ratios (OR, 1.59; 95% CI, 1.40–1.81).

The present study compares the risk of depression and anxiety in people with vision, hearing and dual sensory difficulties in a population of Spanish adults. The data agree with previous literature by confirming the increased risk of depression and anxiety in people with difficulties in seeing and hearing. It adds to the literature through demonstrating the increased risk in those who have both seeing and hearing difficulties, being the first paper to examine and compare single sensory difficulty with dual sensory difficulties for both anxiety and depression.

It is likely that the association between sensory impairment and mental health is bidirectional, and this bidirectionality could be explained by several factors associated to both sensory impairment and mental health. It is likely that that depression in people with difficulties in seeing and hearing may have an underlying etiology involving somatization. Somatization is common in depression, and this could influence the perception of vision and hearing difficulties (Wan et al., 2016). In people with difficulties with seeing, symptoms may be aggravated by other related symptoms such eye discomfort, foreign body sensation and pain. These may contribute to increased disability which in turn could lead to an increase in depression and anxiety. In people with hearing difficulties, depression and anxiety may be aggravated by linked symptoms such as pain or lack of orientation. Difficulties in vision and hearing would also negatively impact the performance of daily activities, emotional well-being, and working capacity. Doctor visits and medical expenses may also contribute. Inability to carry out daily activities that reduces the quality of key lifestyle behaviors, such as socializing with others, physical activity and good diet, known to have protective effect against depression and anxiety (Smith et al., 2017; Jones and Bartlett, 2018; López-Sánchez et al., 2019; Smith et al., 2019), may also play a big role. In addition, the ongoing and progressive nature of the underlying causes that affect vision and hearing may induce and aggravate the depressive and anxiety symptoms. Participants may also underreport depression and/or anxiety because of the relatively low social acceptability of these disorders. It is also possible that aged patients are especially sensitive to negative feelings of helplessness which then increases the risk of depression.

Targeted assessment and treatment of somatic and positive affect symptoms in older adults with visual and hearing difficulties might improve the wellbeing of people with hearing and vision difficulties (Cosh et al., 2020). Anticipating the fact that vision and/or hearing is deteriorating and how rapidly and their effect on the quality of life are important parameters to be considered in order to improve the patient’s mental status. Depressive symptoms may not only aggravate symptoms of sensory difficulties but also affect other psychological systems, for example, feelings of sadness, pessimisms and pain all of which then forms a vicious circle.

It may be appropriate to determine first whether assistive devices such as spectacles and hearing aids can address the visual and hearing difficulties. In 2017, a Lancet Commission report highlighted that hearing losses carried a greater risk of dementia than other potential risk factors such as hypertension and obesity (Livingston et al., 2020). Research suggests that hearing aids, would facilitate greater social engagement, decrease levels of effort required to recognize sounds and speech, decrease levels of depression or anxiety, increase levels of physical balance, and promote greater feelings of independence and self-efficacy (Mulrow et al., 1992; Joore et al., 2002; Chou et al., 2011; Ciorba et al., 2012). If in people with irreversible loss of vision or hearing difficulties, hearing aids and spectacles do not help, then cognitive-behavioral therapy (CBT) or treatment with medication would benefit.

The analysis of covariates in the present study also showed that the population groups with a significantly higher risk of depression and chronic anxiety were females, widowed/divorced/separated, those not living as a couple, those with no tertiary education, those who never smoked, those who did not drink alcohol in the last 12 months, those with obesity, people doing less physical activity, those with cataracts and those with glasses or contact lenses. These results are comparable to those found in previous studies (Bonnet et al., 2005; Zoeller, 2007; Acar et al., 2011; Mendieta Mayorga, 2012; Fazzi et al., 2015; McCusker and Koola, 2015; López-Sánchez et al., 2019) and indicate that depression and anxiety are complex disorders that are influenced by many variables. It is therefore important that that the high prevalence of depression and anxiety in Spain is addressed from a multidisciplinary perspective involving professionals with different expertise in healthcare.

The large representative sample of Spanish adults and analysis of seeing and hearing difficulties in the same population are clear strengths of the present study. However, findings of the present study must be interpreted in light of the studies limitations. First, the data are self-reported potentially introducing self-reporting and recall bias into the findings. Second, the study is cross-sectional and thus it is difficult to know whether the reported sensory impairment increases one’s risk of anxiety and depression or whether the state of mind increases one’s perception of sensory difficulty. Finally, as the stem question for anxiety and depression was “have you ever,” it is possible that some participants suffered from anxiety and depression before the existence of their sensory impairment, which suggests bi-directionality.

## Conclusion

In conclusion, in this large representative sample of Spanish adults, vision difficulty was associated with higher odds of anxiety and depression when compared to hearing difficulty, and importantly a dual sensory difficultly showed the greatest risk. Our results highlight the need for appropriate interventions which may include multidisciplinary programs to improve the mental health status in people with sensory losses. The detection of both sensory difficulty and associated depression represents a priority for clinicians and patients alike. More efforts are needed to identify relevant factors inducing depression and depressive symptoms among people with vision and hearing difficulties and to provide appropriate prevention and treatment for mental disorders.

## Data Availability Statement

The raw data supporting the conclusions of this article will be made available by the authors, without undue reservation.

## Ethics Statement

Ethical review and approval was not required for the study on human participants in accordance with the local legislation and institutional requirements. Written informed consent to participate in this study was provided by the participants’ legal guardian/next of kin.

## Author Contributions

All authors listed have made a substantial, direct and intellectual contribution to the work, and approved it for publication.

## Conflict of Interest

The authors declare that the research was conducted in the absence of any commercial or financial relationships that could be construed as a potential conflict of interest.

## Footnotes

1 https://www.who.int/topics/obesity
